# Radical resection of a giant rib osteosarcoma with complex chest wall reconstruction

**DOI:** 10.1016/j.ijscr.2019.07.080

**Published:** 2019-08-08

**Authors:** Sarwat B. Ahmad, Jason Hoellwarth, Neil Christie, Richard Mcgough

**Affiliations:** a Division of Surgical Oncology, Department of Surgery, University of Pittsburgh School of Medicine, 5230 Centre Ave, Pittsburgh, PA 15232, USA; b Department of Orthopedic Surgery, University of Pittsburgh School of Medicine, 5230 Centre Ave, Pittsburgh, PA 15232, USA; c Division of Thoracic Surgery, University of Pittsburgh School of Medicine, 5230 Centre Ave, Pittsburgh, PA 15232, USA

**Keywords:** Primary rib osteosarcoma, Chest wall reconstruction, Polymethyl methacrylate

## Abstract

**Introduction:**

Primary rib osteosarcoma is a rare chest wall tumor with variable presentation. Large tumors greater than 10 cm are even rarer and present a challenge for surgical management.

**Presentation of case:**

A 61-year-old male with a giant osteosarcoma of the left 2^nd^ rib underwent multidisciplinary management including induction therapy with doxorubicin and cisplatin, followed by en bloc resection with left ribs 1–5, spinous processes of ribs 2–5, small volume lung resection, and chest wall reconstruction with polypropylene mesh and poly methyl methacrylate (PMMA or bone cement). There were no perioperative complications. At 6 months follow-up, the patient remains disease-free. Functional and cosmetic outcome are excellent.

**Discussion:**

This 20 cm mass and resection of ribs 1–5 with resulting 25 cm chest wall defect is the largest primary rib osteosarcoma reported in literature. An R0 resection and chest wall reconstruction using polypropylene mesh and bone cement was feasible and safe.

**Conclusion:**

Giant chest wall defects involving multi-rib resection can be effectively reconstructed with commonly available and inexpensive polypropylene mesh and PMMA to achieve good cosmetic and functional outcomes.

## Highlights

• A 61-year-old male with a 20 cm primary osteosarcoma of the rib underwent a radical en bloc chest wall resection.
• Chest wall reconstruction was performed using a poly methyl methacrylate (“bone cement”) and polypropylene mesh “sandwich”.
• Functional, cosmetic, and oncologic outcomes at 6 months are excellent.
• Polypropylene mesh and “bone cement” represent a safe, easy, and affordable approach to reconstructing the rib cage even in cases of large defects.

## 1 Introduction

Primary osteosarcoma of the rib (POSR) is extremely rare, representing 1.25% of all cases of primary osteosarcoma [1]. Presentation varies based on age, nature and location of the tumor, ranging from minor discomfort to respiratory decompensation. The management of POSR typically includes wide excision and chemotherapy [[2], [3], [4]]. To reduce the risk of relapse, it is important to obtain negative resection margins [5], which can be challenging in the case of large or locally advanced chest wall sarcomas that require multi-rib resection. Several techniques have been employed to reconstruct the chest wall after a radical resection. In line with the SCARE criteria for case reports [6], we describe one of the largest resections to date for POSR requiring skeletal reconstruction with poly methyl methacrylate (PMMA, “cement”), along with a discussion of various techniques used for chest wall reconstruction.

## 2 Presentation of case

**Fig. 1:**
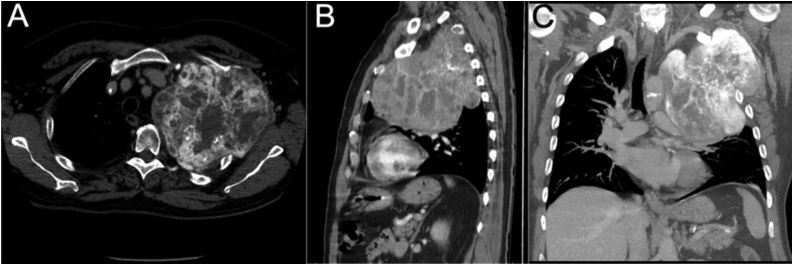
Initial pre-therapy CT images of the thorax in A) axial, B) sagittal, and C) coronal views.

A 61-year-old male was referred to a university orthopaedic practice with left shoulder pain and dyspnea for one year. His medical history included hypertension, coronary artery disease, and 42-pack year smoking history (quit 3 years ago). Physical examination revealed decreased left-sided breath sounds and swelling of the left upper extremity. Neurological exam was normal. Work-up included a chest x-ray followed by chest computed tomography (CT). Representative slices are shown in Fig. 1, revealing a large mass filling the proximal half of the left lung cavity causing tracheal deviation and lung compression into the hilum. The mass featured bony and soft tissue features and measured 15.0 × 16.7 × 15.0 cm. CT-guided core needle biopsy of the mass was consistent with osteosarcoma (Ki-67 10–15%). Bone scan showed no distant disease. A multidisciplinary consensus was obtained, and he was treated with two cycles of doxorubicin and cisplatin prior to surgery. A repeat CT scan showed slight shrinkage in size and increased calcification.

**Fig. 2:**
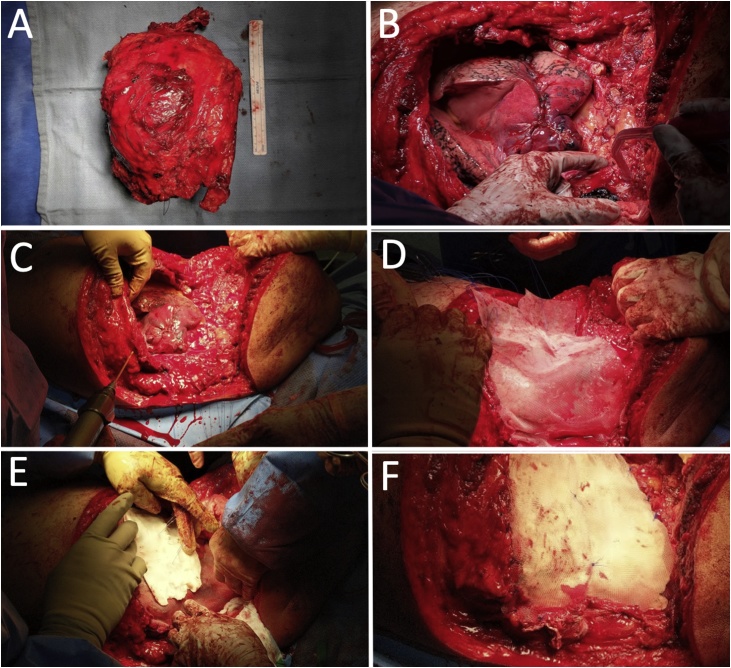
Intraoperative images of A) resected specimen consisting of tumor and ribs 1–5 adjacent to a 15 cm ruler, B) approximately 25 × 25 cm defect in L chest wall, C) drilling into rib for anchoring of mesh, D) placement of inner mesh layer, E) placement of bone cement (PMMA), F) outer mesh layer.

In the operating room, the patient was positioned left-side-up in lateral decubitus. A posterolateral thoracotomy incision was made from left nipple to tip of scapula. The mass was dissected away from the subcutaneous tissue ensuring a soft tissue margin circumferentially. The mass originated from the 2^nd^ rib and involved ribs 1–5 laterally and posteriorly. The mass was adherent to the lung, requiring a stapled wedge resection of a small portion of the left lower lobe. Superiorly, the left subclavian vessels were identified and preserved. Medially, the mass was dissected away from all major mediastinal structures. Ribs 1–5 were cut at the level of the sternum. Posteriorly, we identified multiple pathologic rib fractures and resected portions of spinal bodies T1–T5 at the transverse processes. The specimen was removed en bloc and oriented (Fig. 2A). The chest wall now had a defect of almost the entire length of ribs 1–5 which was approximately 25 × 25 cm (Fig. 2B). Three 36-French chest tubes were placed and secured to skin.

To reconstruct the chest wall, a large piece of polypropylene mesh (Bard Mesh by Davol, Warwich, RI, USA) was first secured circumferentially to bony margins using interrupted non-absorbable sutures. The sutures were placed into the sternum medially, the vertebral bodies posteriorly, and rib 6 inferiorly by either driving the needle through the anchoring bone directly or creating pilot holes with a drill and 2.5 mm drill bit through which suture was passed (Fig. 2C). The mesh was cut to appropriate size and shape, allowing space for lung expansion and an open thoracic outlet (Fig. 2D). Next, a layer of poly methyl methacrylate (PMMA, Simplex HV Cement with Gentamicin by Stryker, Kalamazoo, MI, USA) was spread over the mesh while in its “sticky” phase, creating a plate approximately 2 mm thick to mimic the general contour of the rib cage (Fig. 2E). To prevent thermal injury to the lung below we continuously irrigated the cement with saline during the curing process. Another polypropylene mesh was placed on top of the cement in a similar manner to the first, completely sandwiching the cement between the meshes (Fig. 2F). Two Jackson-Pratt drains were laid over the mesh repair. The subcutaneous flaps were closed in layers over the drains. All chest tubes were placed to −20 mmHg suction. He tolerated the procedure well without complication. Intraoperative blood loss was 1600 ml. He received 4 units of packed red blood cells.

**Fig. 3:**
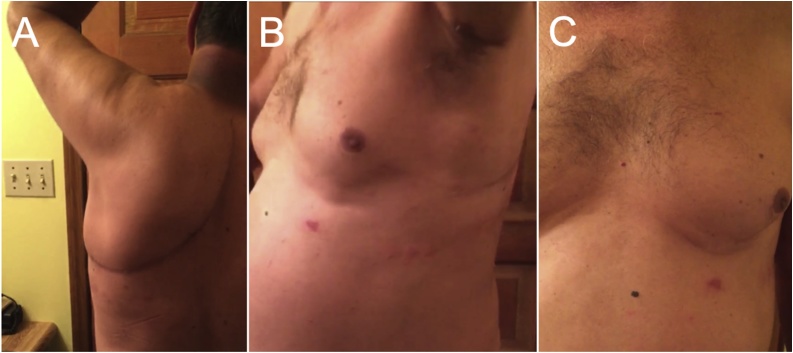
6-month follow-up showing A) well healed incision with intact shoulder abduction, B) chest wall symmetry at rest and C) inspiration.

His postoperative course was unremarkable. Hospital stay was 10 days. He was extubated on day 1. Chest tubes were removed sequentially when appropriate. The surgical drains were removed prior to discharge. He required no oxygen upon discharge. Motor and sensory function in the left upper extremity was intact. The arm was maintained in a sling and permitted passive range of motion. Final pathology confirmed osteogenic sarcoma, negative surgical margins, and 40–50% tumor necrosis indicative of treatment response. All resected lung wedges were negative for malignancy. At 1-month follow-up, he had a well healed incision, normal left upper extremity exam, and nearly symmetric appearance of the thoracic chest wall at rest and with respirations **(**Fig. 3). He completed adjuvant chemotherapy with cisplatin for 4 more cycles and doxorubicin only for cycles 5 and 6 to minimize toxicity. At 6-months, he remains disease-free and pain-free. Right shoulder range of motion continues to improve with physical therapy. Active surveillance will continue every three months with physical exam and CT imaging with or without positron emission tomography to detect recurrence.

## 3 Discussion

The majority of primary osteosarcomas arise in the long bones of children and young adults; thus, primary osteosarcoma of the rib in a middle-aged adult with no prior history of chemotherapy, radiation, or intrinsic bone disease is a rare scenario. We identified fewer than 40 reported cases of POSR since 1980, with a variety of techniques used for chest wall reconstruction.

Defects less than 5 cm and/or located in the dorsoapical or posterior aspect of the chest wall may not require reconstruction [7,8]. However, anterolateral and sternal defects should undergo reconstruction to protect the underlying structures and avoid paradoxical respiration [9]. In bridging the gap between the resected rib ends, the goals of reconstruction include stability, cosmesis, and ensuring an adequate seal to the pleural cavity [10]. Materials are often used in combination to provide an inner layer to protect the viscera, and outer layer for structural support. The various meshes that have been used include: vicryl, Marlex, prolene, and polytetrafluoroethylene (PTFE, Gore-tex).

For rigid support and stabilization, the following materials have been used: titanium plates (e.g. MatrixRIB) [11]; acrylic cement and bone cement [12]; fascia lata grafts [13]; metal rods, struts, and fiberglass. The ideal material combination would allow tissue ingrowth without adherence to underlying viscera, resist infection, and allow shaping to the appropriate conformation. Currently, the maximum rib-rib defect that can be bridged with the titanium MatrixRib plating system is 14 cm [14], and the cut rib ends should allow placement of at least three screws. In our case, the ribs were disarticulated posteriorly and anteriorly, rendering rib plating not a feasible option.

We chose PMMA because it is easily contoured, readily available, inexpensive, and we are familiar with its use and prior success in both the trauma and oncologic settings [15]. PMMA allows more mobility than metal [15]. Preservation of the thoracic curvature and can be easily removed if necessary. Disadvantages include difficulties with anchoring and fixation [14] and potential for infection and device failure as is inherent to any implanted device. We anticipate a low risk of infection as the surrounding mesh incorporates into the soft tissue [16].

In our literature review, the largest reported POSR was 20 × 15 cm requiring resection of ribs 5, 6, and 7, which was reconstructed with a latissimus flap [17]. Lim et al. resected a 9 × 13 cm mass with five contiguous ribs (ribs 3–7) and reconstruction with titanium mesh [18]. In the current case, the tumor size was estimated at 15.0 × 16.7 × 15.0 cm on pre-operative imaging and measured 20.5 × 13.2 × 11.7 cm after resection, involving resection of ribs 1–5, which to our knowledge is one of the largest POSR resections that involved multirib reconstruction. It is thus a valuable addition to the literature and demonstrates surgical feasibility.

## 4 Conclusion

Optimal management of chest wall defects after surgery is a complicated decision that should consider defect size, available anatomic attachment sites for mesh, protection needed for underlying viscera, avoidance of paradoxical respiration, and also the cost and availability of reconstructive materials. The polypropylene mesh and PMMA technique described here offers a medically safe option for small or large chest defects, involves commonly available and relatively low-cost materials, demands a short learning curve using techniques familiar to most general and orthopedic surgeons, and allows immediate patient mobilization without behavior modification while providing long term structural integrity.

## Declaration of Competing Interest

None.

## Sources of funding

None.

## Ethical approval

This case report is exempt from approval by the University of Pittsburgh Institutional Review Board as it includes fewer than three patients.

## Consent

Written informed consent was obtained from the patient for publication of this case report and accompanying images. A copy of the written consent is available for review by the Editor-in-Chief of this journal on request.

## Author’s contribution

Sarwat Ahmad – study concept, writing the paper.

Jason Hoellwarth – study concept, data collection (images), writing the paper.

Neil Christie – study concept and design, editing the paper.

Richard Mcgough – study concept, writing the paper, interpretation of collected data in discussion.

## Registration of research studies

Not applicable.

## Guarantor

Sarwat Ahmad.

Richard McGough.

## Provenance and peer review

Not commissioned, externally peer-reviewed.
